# Laparoscopic (L-) versus Robotic Assisted (RA-) Roux-en-y Gastric Bypass (RYGB): effects on lung function, respiratory muscle strength, and physical activity after six months

**DOI:** 10.1007/s11701-026-03641-w

**Published:** 2026-07-20

**Authors:** Arieli Luz Rodrigues Baretta, Giorgio Alfredo Pedroso Baretta, Verônica Barros, Rafael Schimidt Feistler, Flávio Panegalli Filho, Carolina Mocellin Ghizoni, Alexandre Coutinho Teixeira de Freitas

**Affiliations:** 1 Clinical Surgery, Universidade Federal do Paraná, PR Curitiba, Brazil; 2 Instituto Giorgio Baretta, Curitiba (PR), Brazil; 3 CEPETI – Centro de Estudos e Pesquisa em Terapia Intensiva, Curitiba, Brazil; 4 Universidade Federal de Santa Maria, Santa Maria, (RS), Brazil; 5 Unimed Curitiba - Sociedade Cooperativa de Médicos, Curitiba, (PR), Brazil; 6 Arieli Luz Rodrigues Baretta Alameda Princesa Izabel, 2559, PR Brazil Curitiba, Alameda Princesa Izabel, 80730-080

**Keywords:** Bariatric surgery, Lung function, Laparoscopic surgery, Robotic-assisted surgery

## Abstract

Obesity is a chronic and multifactorial disease associated with systemic changes and pulmonary impairment. Roux-en-Y gastric bypass (RYGB) is effective in treating severe obesity and has been associated with improvements in pulmonary function; however, evidence comparing the effects of laparoscopic (L) and robotic-assisted (RA) RYGB on respiratory outcomes remains limited. To compare the effects of L- and RA- RYGB on lung function, respiratory muscle strength and physical activity level, at baseline and six months postoperatively. Prospective, non-randomized study conducted at a private bariatric surgery center in Brazil, including patients of both sexes, aged 18–65 years with body mass index (BMI) 40.0–49.9 kg/m² undergoing Roux-en-Y gastric bypass. Patients with a history of smoking or pre-existing pulmonary disease were excluded. Patients were divided into L- and RA- groups and evaluated at baseline and six months after surgery with spirometry (forced vital capacity [FVC], forced expiratory volume in one second [FEV₁], and FEV₁/FVC ratio), manovacuometry (maximal inspiratory pressure [MIP] and maximal expiratory pressure [MEP]), and physical activity level (International Physical Activity Questionnaire–Short Form [IPAQ-SF]), following ATS/ERS standards. Statistical analyses included chi-square, Fisher’s exact, Student’s t, Mann-Whitney U, and mixed-effects models (*p* < 0.05). A total of 98 patients were included (49 L-, 49 RA-), with 84 completing the six-month follow-up. Among those who completed follow-up, most participants were women (75.0%), with a mean age of 37.3 years. Significant improvements in pulmonary function were observed over time following surgery. Although a reduction in maximal expiratory pressure was observed in the laparoscopic group in within-group analyses, mixed-effects models showed no significant differences between surgical approaches regarding respiratory muscle strength, pulmonary function, physical activity level, or longitudinal changes over time. Laparoscopic and robotic-assisted Roux-en-Y gastric bypass were associated with improved pulmonary function six months after surgery, with no significant differences between surgical approaches in respiratory outcomes or physical activity level.

**Supplementary Information:**

The online version contains supplementary material available at 10.1007/s11701-026-03641-w.

## Introduction

Obesity is a multifactorial disease associated with systemic inflammation, hormonal changes, and functional impairment. It is considered a chronic, progressive condition and is currently recognized as a global epidemic. Furthermore, obesity has deleterious effects on lung function through multiple interconnected mechanisms, favoring the development of respiratory disorders such as obstructive sleep apnea, hypoventilation syndrome, and asthma [1–5].

Given its increasing prevalence and the difficulty in maintaining weight loss with conservative approaches, bariatric surgery has become one of the most effective strategies for managing severe obesity and associated comorbidities [6–8], including improving lung function and respiratory disorders [9].

Recently, the introduction of the robotic platform represented a technological advancement in minimally invasive surgery, providing greater precision and ergonomics to the surgeon. However, despite the increasing use of robotic-assisted bariatric surgery, the potential benefits of this technology on functional and respiratory recovery remain uncertain, and direct comparisons between robotic and laparoscopic Roux-en-Y gastric bypass are still scarce. Current studies focus predominantly on perioperative outcomes, such as operative time, complications, and costs [10, 11].

In this context, the present study aims to compare the effects of gastric bypass performed by video laparoscopy and robotic-assisted approaches on lung function, respiratory muscle strength and physical activity level, assessed preoperatively and six months after the procedure.

## Methods

This study was submitted to the UFPR Ethics Committee and approved under number 5.690.936.

Data were collected from individuals undergoing gastric bypass surgery at a private bariatric and robotic surgery institute.

Patients aged 18 to 65 years, of both sexes with morbid obesity (BMI 40.0–49.9 kg/m²), who were able to understand the study procedures and who voluntarily agreed to participate in the study by signing an informed consent form, were included. Exclusion criteria comprised extremes of age (under 18 and over 65), those with grade II obesity (BMI < 40 kg/m²) and superobese (BMI > 49.9 kg/m²), smokers, those with prior lung disease, those unavailable for the research protocol, or those who did not sign an informed consent form. Participants were consecutively assigned to two non-randomized groups, with data collected prospectively.

**- Laparoscopic group (L-)**: patients undergoing bariatric surgery via video laparoscopy.
**-Robotic-assisted group (RA-)**: patients undergoing bariatric surgery using the da Vinci X Robotic Platform (da Vinci Surgical System).

All procedures followed the same standardized Roux-en-Y gastric bypass technique and institutional perioperative protocol, differing only in the surgical platform used (laparoscopic or robotic-assisted). All laparoscopic and robotic-assisted procedures were performed by the same bariatric surgeon, thereby minimizing variability related to surgical technique and operator experience.

Participants were evaluated by a single researcher who performed all assessment procedures. The following data were collected: pulse oximetry, vital signs and other demographic characteristics, education level, comorbidities, pulmonary function testing, and respiratory muscle strength. Because of the nature of the surgical interventions, blinding was not feasible. However, all assessments were conducted using standardized protocols by the same researcher, minimizing inter-observer variability and ensuring consistency in data collection. The evaluator was not involved in patient selection, surgical indication, or surgical procedures, which were performed exclusively by the bariatric surgeon. Information regarding comorbidities was obtained from clinical records and preoperative evaluations and was recorded based on documented diagnoses at the time of study enrollment. During the late postoperative assessment, demographic data, comorbidities, pulmonary variables, and physical activity were reassessed. The first evaluation for both groups was conducted on the day prior to hospital admission by the operating surgeon, and the second evaluation was performed at the standardized six-month postoperative follow-up.

Pulmonary function was assessed by spirometry and respiratory muscle strength by manovacuometry, following the technical guidelines of the American Thoracic Society (ATS) and the European Respiratory Society (ERS) [12], ensuring standardized procedures and valid data collection. Both tests were performed with participants seated at a 90-degree angle, feet supported, and wearing a nasal clip.

### Sample size calculation

A sample size calculation was performed during the study design phase based on the primary spirometric outcomes (FVC, FEV1, and FEV1/FVC ratio), considering a significance level of 5% and statistical power of 90%. The largest estimated sample size was obtained for the FEV1/FVC ratio, requiring 84 participants (42 per group). To account for an anticipated loss to follow-up of 15–20%, a total of 100 participants (50 per group) was planned for recruitment.

### Spirometry

A Spirobank II Advanced^®^ spirometer, certified by the Brazilian Ministry of Health (Anvisa registration) and compliant with ATS/ERS 2019 guidelines, was used [12]. Forced expiratory volume in one second (FEV₁), forced vital capacity (FVC) and the FEV₁/FVC ratio were measured. Participants were instructed to avoid using short-acting bronchodilators in the hours before the exam. Spirometry tests with at least three acceptable and two reproducible maneuvers were considered valid. Results were expressed in absolute and percentage values of the expected, based on the Global Lung Function Initiative [13] equations, adjusted for gender, age, height, weight and ethnicity.

### Manovacuometry

Manovacuometry was performed through a HOMED^®^ brand digital manovacuometer, with a measurement range of **-300 to + 300 cmH₂O**, in accordance with ATS/ERS 2019 technical criteria [12]. Maximum inspiratory pressure (MIP) was evaluated from residual volume, by maximum inspiratory effort against an occluded airway, while the maximum expiratory pressure (MEP) was obtained from total lung capacity through maximum expiratory effort. For each measurement, three to five maneuvers were performed; valid maneuvers were defined as those sustained for at least one second with a difference of less than 10% between the two highest values. The highest reproducible value was considered for analysis.

Participants’ physical activity levels were assessed using the International Physical Activity Questionnaire – Short Form (IPAQ-Short Form), an internationally validated instrument recommended by the World Health Organization (WHO) for measuring physical activity in adult populations [14]. The questionnaire was administered in person by the researcher, and responses recorded according to the reference period of the previous seven days. Participants were classified according to IPAQ physical activity categories.

### Statistical analysis

Statistical analyses were performed using IBM SPSS Statistics software, version 29.0 (IBM Corp., Armonk, NY, USA). Categorical variables were described as absolute and relative frequencies and compared using Pearson’s chi-square test, Fisher’s exact test, or Fisher–Freeman–Halton exact test, when appropriate. Continuous variables were expressed as mean ± standard deviation. Normality was assessed through graphical inspection and evaluation of distributional assumptions.

Baseline comparisons between the laparoscopic (L-) and robotic-assisted (RA-) groups were performed using Student’s t-test for independent samples when normality assumptions were met and the Mann–Whitney U test otherwise.

To evaluate potential attrition bias, baseline demographic, anthropometric, clinical, and respiratory characteristics were compared between participants who completed the 6-month follow-up and those lost to follow-up using the same statistical procedures.

Longitudinal changes in weight, BMI, pulmonary function, and respiratory muscle strength were evaluated using mixed-effects models including group (L- vs. RA-), time (baseline vs. 6 months), and the group × time interaction term. The interaction term was considered the primary test of whether changes over time differed according to surgical approach.

For descriptive purposes, within-group comparisons between baseline and 6-month assessments were additionally performed using paired Student’s t-tests or Wilcoxon signed-rank tests, according to data distribution.

All tests were two-tailed, and p-values < 0.05 were considered statistically significant.

## Results

Data were collected from 98 patients undergoing gastric bypass. Of these, 49 underwent surgery via video laparoscopy (L- group) and 49 via robotic surgery (RA- group). At the six-month postoperative assessment, 84 patients (85.7%) returned: 43 from the video laparoscopy group and 41 from the robotic surgery group.

An attrition bias analysis comparing participants who completed the 6-month follow-up (*n* = 84) with those lost to follow-up (*n* = 14) is presented in Supplementary Table S1. Most baseline demographic, anthropometric, clinical, and pulmonary characteristics were comparable between groups. However, participants lost to follow-up had a higher prevalence of hepatic steatosis and higher baseline maximal expiratory pressure (MEP) than those who completed the study.

The groups were homogeneous in most preoperative characteristics, differing significantly only in educational level. The characteristics of this sample that completed follow-up are described in Table 1.

**Table 1:** Demographics of L- and RA- groups

Variables	L- group* (*n* = 43)	RA- group* (*n* = 41)	*P*-value
**Sex**			> 0.999
Female	32 (74.4%)	31 (75.6%)	
Male	11 (25.6%)	10 (24.4%)	
**Age (years)**	35.7 ± 7.7	38.9 ± 9.9	0.104
**Race**			0.558
Black	3 (7%)	2 (4.9%)	
White	37 (86%)	38 (92.7%)	
Asian	3 (7%)	1 (2.4%)	
**Schooling**			0.037
Completed Elementary or High School	9 (20.9%)	6 (14.6%)	
Incomplete higher education	11 (25.6%)	3 (7.3%)	
Completed higher education	23 (53.5%)	32 (78%)	
**Preoperative Comorbidity**	35 (81.4%)	31 (75.6%)	0.600
Arterial hypertension	11 (25.6%)	12 (29.3%)	0.808
Diabetes Mellitus	1 (2.3%)	2 (4.9%)	0.611
Dyslipidemia	4 (9.3%)	5 (12.2%)	0.735
Arthropathy	8 (18.6%)	10 (24.4%)	0.600
Obstructive sleep apnea	6 (14%)	13 (31.7%)	0.069
Gastroesophageal reflux disease	11 (25.6%)	15 (36.6%)	0.347
Fatty liver (hepatic steatosis)	23 (53.5%)	17 (41.5%)	0.285
Cholelithiasis	1 (2.3%)	4 (9.8%)	0.197
Other comorbidities	5 (11.6%)	3 (7.3%)	0.713
**Height (m)**	1.7 ± 0.1	1.7 ± 0.1	0.661
**Weight (kg)**	115.6 ± 11.6	117.3 ± 14.1	0.925
**BMI (kg/m** ^**2**^ **)**	41.6 ± 2.3	41.9 ± 2.7	0.872
**Obesity Type**			0.588
Central	12 (27.9%)	13 (31.7%)	
Peripheral	1 (2.3%)	0 (0%)	
Mixed	30 (69.8%)	28 (68.3%)	
**Spirometry**			
Preoperative FVC	3.9 ± 0.7	3.9 ± 0.7	0.996
% of predicted preoperative FVC	100.3 ± 15.2	103.2 ± 13.8	0.519
Preoperative FEV_1_	3.3 ± 0.6	3.2 ± 0.5	0.450
% of predicted preoperative FEV_1_	97.3 ± 15.6	98.2 ± 12.7	0.975
Preoperative FEV_1_/FVC	82.1 ± 13.9	81.3 ± 7.4	0.202
% of predicted preoperative FEV_1_/FVC	100.5 ± 9.3	97.8 ± 9.7	0.316
**Manovacuometry**			
Preoperative MIP	98.6 ± 36.5	92.3 ± 29.3	0.457
Preoperative MEP	86 ± 32.8	85.4 ± 30.4	0.609

* Categorical variables were described by frequency (percentage) and quantitative variables by mean ± standard deviation

Comparisons of preoperative and 6-month postoperative weight, BMI, pulmonary function, and respiratory muscle strength are presented in Table 2 for the laparoscopic group and in Table 3 for the robotic-assisted group.

**Table 2:** Primary and secondary outcomes of L- group

Variables	Baseline	6 months	*P*-value
**Weight (kg)**	115.6 ± 11.6	80.1 ± 10.8	< 0.001
**BMI (kg/m** ^**2**^ **)**	41.6 ± 2.3	28.8 ± 2.8	< 0.001
**Spirometry**			
FVC	3.9 ± 0.7	4.0 ± 0.8	0.004
% of predicted FVC	100.3 ± 15.2	104.8 ± 12.7	0.001
FEV_1_	3.3 ± 0.6	3.4 ± 0.6	< 0.001
% of predicted FEV_1_	97.3 ± 15.6	102.7 ± 13.7	< 0.001
FEV_1_/FVC	82.1 ± 13.9	82.3 ± 15.2	0.741
% of predicted FEV_1_/FVC	100.5 ± 9.3	98.5 ± 18.1	0.558
**Manovacuometry**			
MIP	98.6 ± 36.5	95.5 ± 32.7	0.425
MEP	86.0 ± 32.8	80.3 ± 31.1	0.033

* Results described by mean ± standard deviation

The data demonstrate a significant reduction in weight and BMI six months after surgery, as well as a significant improvement in spirometric parameters: FVC and FEV_1_ (Tables 2 and 3).

**Table 3:** Primary and secondary outcomes of RA group

Variables	Baseline	6 months	*P*-value
**Weight (kg)**	116.4 ± 14.0	82.0 ± 13.1	< 0.001
**BMI (kg/m** ^**2**^ **)**	41.9 ± 2.7	29.6 ± 4.1	< 0.001
**Spirometry**			
FVC	3.9 ± 0.7	4.2 ± 0.8	< 0.001
% of predicted FVC	103.2 ± 13.8	109.7 ± 13.4	< 0.001
FEV_1_	3.2 ± 0.5	3.4 ± 0.6	< 0.001
% of predicted FEV_1_	98.2 ± 12.7	104.6 ± 13.5	< 0.001
FEV_1_/FVC	81.3 ± 7.4	81.6 ± 13.3	0.480
% of predicted FEV_1_/FVC	97.8 ± 9.7	98.1 ± 16.1	0.863
**Manovacuometry**			
MIP	92.3 ± 29.3	92.1 ± 30.1	0.993
MEP	85.4 ± 30.4	84.5 ± 25.3	0.625

* Results described by mean ± standard deviation

Regarding manovacuometry, a significant reduction in MEP was observed in the L- group six months after surgery, while no significant change in MIP was noted in this group (Table 2). Conversely, in the RA-group, no significant changes were observed in any of the manovacuometric parameters six months after surgery (Table 3).

The results of the intergroup comparative analysis of variables representing lung function, weight, BMI, presence of comorbidities, and physical activity level classified by the IPAQ obtained at the 6-month post-operative assessment are shown in Table 4. No significant differences were observed between the groups, consistent with the findings from the preoperative assessment.

**Table 4:** Comparison of six-month postoperative outcomes between the laparoscopic and robotic-assisted groups

Variables	L- group *(*n* = 43)	RA- group (*n* = 41)	*P*-value
**Weight (kg)**	80.1 ± 10.8	82.0 ± 13.1	0.749
**BMI (kg/m** ^**2**^ **)**	28.8 ± 2.8	29.6 ± 4.1	0.274
**Spirometry**			
FVC	4.0 ± 0.8	4.2 ± 0.8	0.237
% of predicted FVC	104.8 ± 12.7	109.7 ± 13.4	0.169
FEV_1_	3.4 ± 0.6	3.4 ± 0.6	0.999
% of predicted FEV_1_	102.7 ± 13.7	104.6 ± 13.5	0.532
FEV_1_/FVC	82.3 ± 15.2	81.6 ± 13.3	0.950
% of predicted FEV_1_/FVC	98.5 ± 18.1	98.1 ± 16.1	0.914
**Manovacuometry**			
MIP	95.5 ± 32.7	92.1 ± 30.1	0.622
MEP	80.3 ± 31.1	84.5 ± 25.3	0.330
**IPAQ**			0.247
Very active	8 (20.5%)	14 (36.8%)	
Active	17 (43.6%)	15 (39.5%)	
Insufficiently active	3 (7.7%)	4 (10.5%)	
Sedentary	11 (28.2%)	5 (13.2%)	

*Continuous variables are presented as mean ± standard deviation, and categorical variables as frequency (percentage)

To further evaluate whether changes over time differed according to surgical approach, mixed-effects models including group (laparoscopic versus robotic-assisted), time (baseline versus 6 months), and the group x time interaction term were performed. Significant effects of time were observed for weight, BMI, percentage of predicted FVC, FEV1, and percentage of predicted FEV1, indicating overall improvement after surgery. However, no significant group x time interactions were identified for any anthropometric, pulmonary function, or respiratory muscle strength variable, demonstrating that the magnitude of change over time was similar between laparoscopic and robotic-assisted RYGB (Table 5).

**Table 5:** Mixed-effects model analysis of anthropometric, pulmonary function, and respiratory muscle strength outcomes

Variables	*p*-value Group	*p*-value Time	*p*-value Group x Time
**Weight (kg)**	0.478	< 0.001	0.932
**BMI (kg/m** ^**2**^ **)**	0.680	< 0.001	0.521
**Spirometry**			
FVC	0.815	0.157	0.409
% of predicted FVC	0.304	0.049	0.567
FEV_1_	0.531	0.046	0.619
% of predicted FEV_1_	0.862	0.018	0.786
FEV_1_/FVC	0.775	0.950	0.987
% of predicted FEV_1_/FVC	0.347	0.479	0.569
**Manovacuometry**			
MIP	0.507	0.700	0.799
MEP	0.977	0.301	0.533

The group × time interaction was considered the primary analysis for assessing differential longitudinal changes between surgical approaches

## Discussion

The literature consistently demonstrates that bariatric surgery is associated with improved lung function, particularly dynamic lung volumes. In a meta-analysis involving more than 1,000 patients, Alsumali et al. (2018) [9] observed a significant increase in forced vital capacity (FVC) and forced expiratory volume in the first second (FEV_1_) after bariatric surgery, with follow-up periods of between three and 12 months. Similar results were described by Qin et al. (2023) [8], who, in a systematic review and meta-analysis involving patients with obesity and obstructive sleep apnea, reported significant improvement in FVC and oxygenation parameters, especially during the first year after the surgical procedure.

However, both studies analyzed different bariatric techniques in a grouped manner, without specific stratification by type of procedure, which limits interpretations about the isolated impact of gastric bypass on lung function.

Although robotic surgery has been progressively incorporated into bariatric practice, the literature comparing laparoscopic and robotic approaches focuses predominantly on perioperative outcomes, such as surgical time, general complications, and reoperation rates. A recent systematic review conducted by Leang et al. (2024) [15] compared gastric bypass performed robotically and laparoscopically, but exclusively evaluated clinical and perioperative safety outcomes, without including systematic assessments of pulmonary function or respiratory muscle strength in the postoperative periods. This absence of comparative respiratory data between the two approaches highlights a significant gap in the literature.

In the present study, both groups demonstrated significant improvement in spirometric parameters, particularly FVC and FEV_1_, six months after surgery, reinforcing the effectiveness of the procedure on pulmonary function in the short term. These findings are consistent with previous studies evaluating pulmonary outcomes after Roux-en-Y gastric bypass during similar postoperative periods. Tu et al. (2015) [16], in a six-month follow-up study after RYGB, also demonstrated significant increases in FVC and FEV_1_ following surgery, although their sample included patients with class I and II obesity, whereas the present study evaluated exclusively patients with class III obesity.

Similar results have also been described in studies involving other bariatric techniques. Şahin et al. (2025) [17] demonstrated an increase in FEV_1_ proportional to the reduction in BMI in patients undergoing vertical sleeve gastrectomy, while Noori et al. (2021) [18] and Umemura et al. (2022) [19] reported improvements in lung volumes and capacities at 12 months postoperatively. Improvements in pulmonary function have also been observed during long-term follow-up after bariatric surgery. Roth et al. (2023) [20] followed patients for up to 10 years after gastric bypass and observed that 73% of those with impaired pulmonary function preoperatively showed normalization of parameters during follow-up. Taken together, these data suggest that the improvement in pulmonary function observed after bariatric surgery reflects favorable adaptations of respiratory mechanics resulting from weight loss, regardless of the surgical technique used.

In line with this interpretation, Abbasi et al. (2025) [21] also observed significant increases in static and dynamic lung volumes after bariatric surgery, with a direct correlation between the magnitude of weight loss and improvement in functional capacity.

In addition, the absence of differences between the laparoscopic and robotic access routes observed in the present study suggests that the surgical approach may not substantially influence pulmonary recovery during the six-month follow-up period.

The comparison between the groups undergoing gastric bypass via laparoscopy and robotic surgery did not demonstrate significant differences in respiratory muscle strength parameters six months after surgery. However, the intragroup analysis revealed a significant reduction in MEP only in the laparoscopic group, without changes in MIP, while the robotic group maintained stable values of respiratory muscle strength throughout the follow-up.

Previous studies suggest that respiratory muscle strength after bariatric surgery may be influenced by multiple factors, including postoperative recovery, physical activity level, and physiotherapy interventions [22–24]. However, specific evidence relating laparoscopic or robotic approaches to differential preservation of respiratory muscle strength after bariatric surgery remains scarce. In the present study, although a reduction in MEP was observed in the laparoscopic group, no significant between-group differences were identified at six months postoperatively. Therefore, the mechanisms underlying this isolated finding remain undefined and should be interpreted with caution. Although statistically significant, the clinical implications of this isolated reduction in PEmax remain uncertain.

Thus, although the reduction in MEP observed in the laparoscopic group represents an exploratory finding, the present data do not allow us to conclude that the surgical approach directly influences respiratory muscle strength, reinforcing the need for randomized studies with matched groups to further elucidate this issue. Given the limited evidence currently available on respiratory outcomes following robotic-assisted bariatric surgery, the present findings provide clinically relevant information by demonstrating comparable respiratory outcomes between laparoscopic and robotic-assisted Roux-en-Y gastric bypass six months after surgery.

### Limitations of the study

Some limitations should be considered when interpreting the results. This is a prospective, but non-randomized study with consecutive allocation of participants, which limits direct causal interpretations between the surgical approach and the outcomes assessed. The absence of formal matching between groups may have contributed to uncontrolled residual variability, despite the similarity of baseline characteristics. In addition, randomization between laparoscopic and robotic-assisted approaches was not feasible, since robotic-assisted bariatric surgery is not routinely covered by private health insurance in Brazil, potentially contributing to selection bias. The six-month follow-up is short-term, not allowing for the assessment of the persistence of respiratory adaptations observed over time.

Although the follow-up rate was high (85,7%), some participants were lost during follow-up. An attrition bias analysis showed that participants lost to follow-up had a higher prevalence of hepatic steatosis and higher baseline maximal expiratory pressure (MEP) than those who completed the study. Therefore, the possibility of residual selection bias related to these characteristics cannot be completely excluded.

Perioperative variables such as postoperative pain, analgesic use, respiratory complications, and hospital stay were not included in the present analysis, since the primary objective of the study was to evaluate respiratory outcomes six months after surgery rather than immediate postoperative recovery.

Despite this, the findings of the present study have relevant clinical implications. The significant improvement in pulmonary function observed as early as six months after gastric bypass reinforces the positive impact of bariatric surgery on respiratory mechanics in the short term, regardless of the approach used. The absence of significant differences between laparoscopic and robotic approaches suggests that the choice of access route, from a respiratory standpoint, can be guided by technical factors, surgeon experience, and resource availability, without compromising pulmonary recovery. Furthermore, the results highlight the importance of postoperative physiotherapy follow-up, especially in monitoring respiratory muscle strength, since subtle changes can occur even in the absence of intergroup differences. The scarcity of studies evaluating comparative respiratory outcomes between minimally invasive surgical approaches reinforces the relevance of the findings presented. Future randomized studies with longer follow-up periods and more comprehensive respiratory assessments are needed to more precisely elucidate the impact of the surgical access route on pulmonary recovery after bariatric surgery.

## Conclusion

Roux-en-Y gastric bypass was associated with significant improvements in pulmonary function six months after surgery. No significant differences were observed between laparoscopic and robotic-assisted approaches regarding spirometric parameters, respiratory muscle strength, physical activity level, or longitudinal changes over time. Although a reduction in maximal expiratory pressure (MEP) was observed in the within-group analysis of the laparoscopic group, mixed-effects models did not demonstrate a significant group × time interaction for respiratory muscle strength outcomes, indicating that respiratory muscle strength evolved similarly in both surgical approaches.

## Supplementary Information

Below is the link to the electronic supplementary material.

Supplementary Material 1

## Data Availability

Data are available upon reasonable request due to privacy/ethical restrictions.

## Declarations

### Research data

Data are available upon reasonable request due to privacy/ethical restrictions.
