# Supplementary material for: Laparoscopic (L-) versus Robotic Assisted (RA-) Roux-en-y Gastric Bypass (RYGB): effects on lung function, respiratory muscle strength, and physical activity after six months
Source: J Robot Surg. 2026 Jul 20;20(1):683. doi: 10.1007/s11701-026-03641-w (PMC13385452; doi:10.1007/s11701-026-03641-w)
Supplement: Supplementary file 1 — Supplementary Material 1 [file 11701_2026_3641_MOESM1_ESM.docx]

**Supplementary Material:**

**Supplementary Table S1. Baseline comparison between participants who completed the 6-month follow-up and those lost to follow-up (attrition bias analysis)**

| **VARIABLES** | **Patients with 6-month follow-up (included in the analysis)* (n=84)** | **Patients lost to follow-up* (n=14)** | **P-value** |
| --- | --- | --- | --- |
| **SEX** |  |  |  |
| Female | 63 (75%) | 8 (57,1%) | 0,200# |
| Male | 21 (25%) | 6 (42,9%) |  |
| **AGE (years)** | 37 ± 9 | 41 ± 10 | 0,159§ |
| **RACE** |  |  |  |
| Black | 5 (6%) | 0 (0%) | 0,843¨ |
| White | 75 (89,3%) | 13 (92,9%) |  |
| Asian | 4 (4,8%) | 1 (7,1%) |  |
| **SCHOOLING** |  |  |  |
| Completed Elementary or High School | 15 (17,9%) | 3 (21,4%) | 0,938¬ |
| Incomplete higher education | 14 (16,7%) | 2 (14,3%) |  |
| Completed higher education | 55 (65,5%) | 9 (64,3%) |  |
| **PRE-OPERATIVE COMORBIDITY** | 66 (78,6%) | 12 (85,7%) | 0,728# |
| Arterial hypertension | 23 (27,4%) | 2 (14,3%) | 0,508# |
| Diabetes Mellitus | 3 (3,6%) | 2 (14,3%) | 0,148# |
| Dyslipidemia | 9 (10,7%) | 1 (7,1%) | 1,0# |
| Arthropathy | 18 (21,4%) | 3 (21,4%) | 1,0# |
| Obstructive sleep apnea | 19 (22,6%) | 3 (21,4%) | 1,0# |
| Gastroesophageal reflux disease | 26 (31%) | 5 (35,7%) | 0,761# |
| Fatty liver (hepatic steatosis) | 40 (47,6%) | 11 (78,6%) | 0,043# |
| Cholelithiasis | 5 (6%) | 2 (14,3%) | 0,261# |
| Other comorbidities | 8 (9,5%) | 0 (0%) | 0,597# |
| **HEIGHT (meters)** | 1,7 ± 0,1 | 1,7 ± 0,1 | 0,974§ |
| **WEIGHT (kg)** | 116,4 ± 12,9 | 118,6 ± 16,4 | 0,726£ |
| **BMI (kg/m^2^)** | 41,7 ± 2,5 | 42,5 ± 3,3 | 0,879£ |
| **OBESITY TYPE** |  |  |  |
| Central | 25 (29,8%) | 9 (64,3%) | 0,056¨ |
| Peripheral | 1 (1,2%) | 0 (0%) |  |
| Mixed | 58 (69%) | 5 (35,7%) |  |
| **SPIROMETRY** |  |  |  |
| Pre-operative FVC | 3,9 ± 0,7 | 3,8 ± 0,8 | 0,781§ |
| % of predicted pre-operative FVC | 102 ± 15 | 97 ± 10 | 0,201§ |
| Pre-operative FEV_1_ | 3,2 ± 0,6 | 3,1 ± 0,6 | 0,582§ |
| % of predicted pre-operative FEV_1_ | 98 ± 14 | 93 ± 11 | 0,203§ |
| Pre-operative FEV_1_/FVC | 81,7 ± 11,2 | 85,9 ± 13,5 | 0,707£ |
| % of predicted pre-operative FEV_1_/FVC | 99 ± 10 | 104 ± 16 | 0,626£ |
| **MANOVACUOMETRY** |  |  |  |
| Pre-operative MIP | 95 ± 33 | 99 ± 39 | 0,651§ |
| Pre-operative MEP | 86 ± 31 | 104 ± 22 | 0,036§ |

* Categorical variables are presented as frequency (percentage), and continuous variables as mean ± standard deviation.

# Significantity of Fisher's exact test

¬Significance of the Chi-square test

¨ Significance of the Fisher-Freeman-Halton Exact Test

§ Significance of the Student's t-test for independent samples

£ Significance of the Mann-Whitney test
